# Silicon Carbide Nanostructures as Potential Carbide Material for Electrochemical Supercapacitors: A Review

**DOI:** 10.3390/nano13010150

**Published:** 2022-12-28

**Authors:** Gunendra Prasad Ojha, Gun Woong Kang, Yun-Su Kuk, Ye Eun Hwang, Oh Hoon Kwon, Bishweshwar Pant, Jiwan Acharya, Yong Wan Park, Mira Park

**Affiliations:** 1 Carbon Composite Energy Nanomaterials Research Center, Woosuk University, Wanju-Gun, Chonbuk 55338, Republic of Korea; 2 Woosuk Institute of Smart Convergence Life Care (WSCLC), Woosuk University, Wanju, Chonbuk 55338, Republic of Korea; 3 Research and Development Division, Korea Institute of Convergence Textile, Iksan, Chonbuk 54588, Republic of Korea; 4 Convergence Research Division, Korea Carbon Industry Promotion Agency (KCARBON), Jeonju, Chonbuk 54853, Republic of Korea

**Keywords:** silicon carbide, supercapacitors, nanoarchitectures, specific capacitance

## Abstract

Silicon carbide (SiC) is a very promising carbide material with various applications such as electrochemical supercapacitors, photocatalysis, microwave absorption, field-effect transistors, and sensors. Due to its enticing advantages of high thermal stability, outstanding chemical stability, high thermal conductivity, and excellent mechanical behavior, it is used as a potential candidate in various fields such as supercapacitors, water-splitting, photocatalysis, biomedical, sensors, and so on. This review mainly describes the various synthesis techniques of nanostructured SiC (0D, 1D, 2D, and 3D) and its properties. Thereafter, the ongoing research trends in electrochemical supercapacitor electrodes are fully excavated. Finally, the outlook of future research directions, key obstacles, and possible solutions are emphasized.

## 1. Introduction

Globally, we are facing numerous threats due to the reduction of natural combustion-based energy resources and high CO_2_ emissions. Thus, it is urgent to develop low carbon emission energy resources [1,2,3,4,5,6,7,8,9,10,11,12]. To date, the storage of energy has exclusively been based on supercapacitors (SCs) and batteries (Li-batteries) [13,14,15,16,17,18]. Due to their high energy density, batteries are the most accepted and adapted candidate [16,19]. However, when massive energy is needed with high power, SCs remain the best choice [20,21,22]. SCs are electrochemical energy storage devices that offer a higher charge storage ability than conventional capacitors with low internal resistance [23,24]. Supercapacitors have several enticing qualities, such as their quick charging ability, high power density, safe operation, cheapness, pronounced rate capability, and wide range of working ability (−70–100 C) [25,26]. However, their energy density is still far beyond Li-batteries, which restricts their proper implementation in the industrial sectors [17,24,27,28]. Thus, most of the researchers and their research works have been devoted to increasing the energy density closer to the Li-batteries [29,30]. The main components of SCs are electrode material, electrolyte, and separator, which electrically separates two electrodes [30,31]. The most crucial and central component of a supercapacitor is the electrode, and the overall performance of an SC is determined by the overall electrochemical activities of its electrode [7,32,33,34]. Depending on the charge storage process, supercapacitors can be categorized in to two types: (i) electric double layer (EDLC), in which the capacitance is realized rapidly at the interface between electrode and electrolyte [8]. Mostly, carbon and its derivatives such as activated carbon, graphene oxide, carbon naontubes, SiC, and carbon nanofibers are widely-employed EDLC materials [5,21,24,30,35,36,37]; and (ii) pseudocapacitors, in which capacitance originates mainly due to the quick faradic redox reaction between electrode and electrolyte [38,39]. Metal-based materials such as oxides, hydroxides, selenides, sulfides, phosphides, and conductive polymers are the major pseudocapacitive materials in this case [2,18,22,23,28,40,41,42,43].

Among the various electrode materials, silicon carbide (SiC) is a unique class of carbide materials in which Si and C atoms are covalently bonded via the sharing of electron pairs in Sp^3^ hybrid orbitals. In comparison with commonly used carbide materials, SiC is one of the most fascinating candidates of the next generation, with a series of potential physiochemical properties such as adjustable band gaps ranging from 2.4 to 3.2 V, a variety of prototype structures (4H, 6H, and 3C) and dimensions (0D, 1D, 2D, and 3D), non-toxicity, high electron mobility, and chemical stability in harsh conditions [44,45,46,47]. These potential physiochemical properties make SiC an interesting candidate as an electrode material for supercapacitor applications. Pure SiC electrodes store charge via the adsorption of electrolyte ions at the electrode and electrolyte interfaces, which makes them ideal for storing high power. However, low varieties of synthetic techniques are being widely used to synthesize nanostructured SiC (0D, 1D, 2D, and 3D) and heteroatoms doped SiC for supercapacitors electrodes. SiC not only acts as an electrode itself but is also used as conductive support to grow different nanostructures, making various composite materials and core-shell structures as electrodes for supercapacitors [48,49]. 

In this review, we present the current research activities that focus on the synthesis and properties of supercapacitor electrodes. The present work is organized into six sections. In Section 1, Section 2, Section 3, Section 4 and Section 5, we offer a brief introduction and then describe the crystallographic structure, synthesis techniques, properties, and applications of electrochemical supercapacitors, respectively. Finally, in Section 6, we conclude our outlooks on challenges and future opportunities. 

## 2. Crystallographic Structure of SiC

In silicon carbide (SiC), a strong covalent bond exists between Si and C (Si-C). More than 250 polytyes of SiC have been reported so far; however, very few of them are studied properly. The crystallographic structures of SiC depend on the close-packed stacking of double-layers of Si and C atoms. There exist different crystallographic forms (i.e. polyporphs) such as 3C, 2H, 4H, 3C, hexagonal 4H and 6H, and rhombohedral R. The red and yellow balls signify the Si and C atoms, and A, C, C, and D represent the stacking positions of C and Si double layers. SiCs possessing a single cubic structure 3C, called beta-SiC 6H and 15R, are found based on their stacking sequences between Si-C–Si-C layers. The frequently used notation of different polymorphs of SiC was first introduced by Ramasdell in 1947 [50]. 

**Figure 1:**
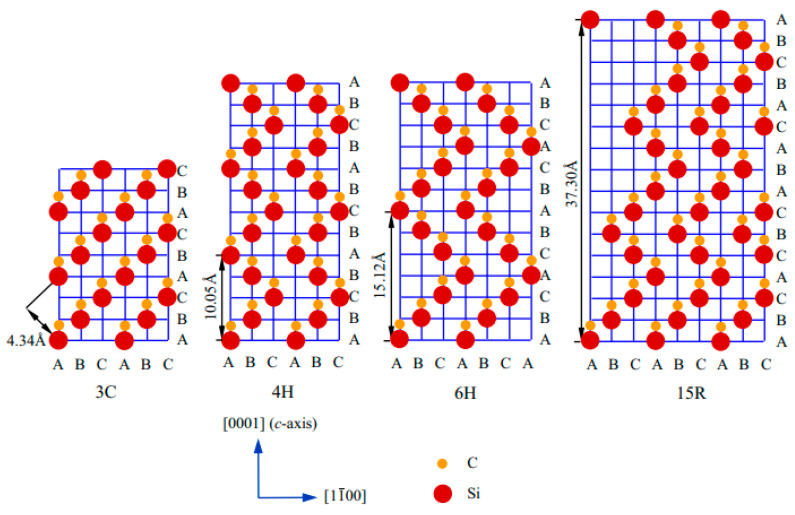
Stacking sequences of layered 3C-, 4H-, 6H-, and 15R-SiC [50].

Figure 1 indicates the arrangement of atoms in the most commonly used polytypes such as cubic 3C, hexagonal 4H and 6H, and rhombohedral R (15R). The red and yellow balls signify the Si and C atoms, and A, C, C, and D represents the stacking positions of C and Si double layers, respectively. SiC has a single cubic structure 3C, called beta-SiC, and the stacking sequence is represented as ABC. The hexagonal and rhombohedral forms are represented as α-SiC. Hexagonal 4H, hexagonal 6H, and rhombohedral 15R are represented as ABCB…, ABCACB…, and ABCACBCABACABBC…, respectively. The nature of the stacking strongly modulates the physiochemical properties such as optical, electronic, and thermal [50].

## 3. Synthesis Process of SiC Nanoarchitectures

In 1994, Zhou et al. successfully synthesized SiC nanowhiskers without using catalysts by reacting silicon dioxide and carbon nanocluster at an elevated temperature of 1700 °C for 2 h in an inert atmosphere [51]. Since then, a huge breakthrough in the synthesis of SiC nanostructures (0D, 1D, 2D, and 3D) has been accomplished.

### 3.1. 0D SiC Nanoarchitectures

**Figure 2:**
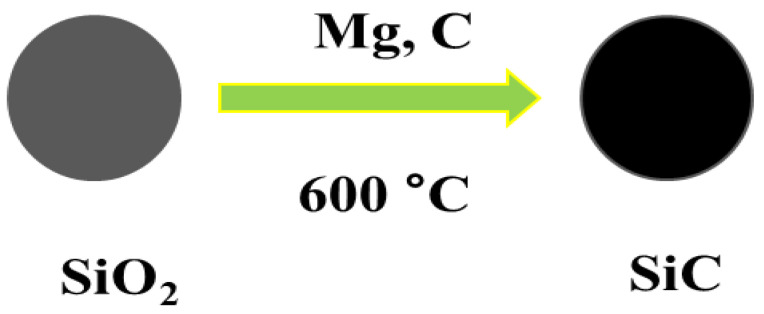
Schematic representation showing the synthesis process of SiC NPs.

**Figure 3:**
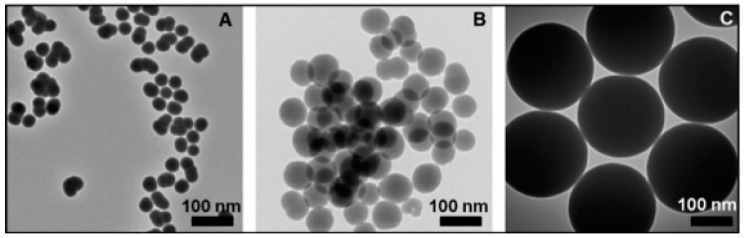
TEM images (**A**–**C**) of SiC NPs at different reaction temperatures [44].

Diverse synthesis approaches have been invented to synthesize 0D nanoarchitecutes such as hollow nanospheres, hollow nanocages, solid nanocrystals, and core and/or shell nanospheres. Zhu et al. synthesized large quantities of β-SiC nanocrystals via a facile chemical etching process. The cubic SiC, handled as a powder, was etched in a mixed solution of 65% nitric acid and 40% hydrofluoric acid (volume ratio = 1:3) at 100 °C for 1 h [52]. Veinot et al. synthesized spherical β-SiC by way of a low temperature solid-state metathesis reaction between SiO_2_, Mg, and C powder [53]. Figure 2 and Figure 3 represents the synthesis process and TEM images.

**Figure 4:**
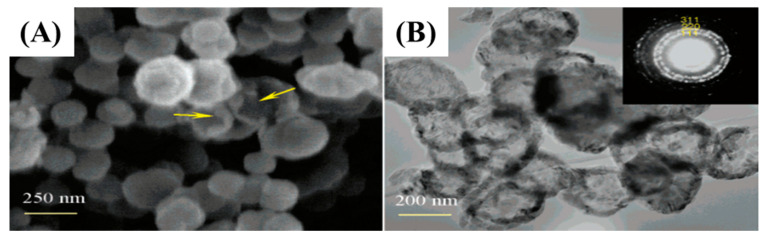
FE-SEM and TEM images (**A**,**B**) of hollow SiC NPs [45].

Similarly, Liu et al. prepared hollow nanospheres through a vapor-solid reaction between SiO and amorphous carbon nanoparticles [54]. Equal amounts of silica- and silicon-mixed powder and amorphous carbon NPs were employed and heated at 1300 °C in an argon atmosphere. At elevated temperatures, the mixture of Si and SiO_2_ generated SiO, which further reacted with C to convert hollow SiC-NPs as shown in Figure 4.

### 3.2. 1D SiC Nanoarchitectures

**Figure 5:**
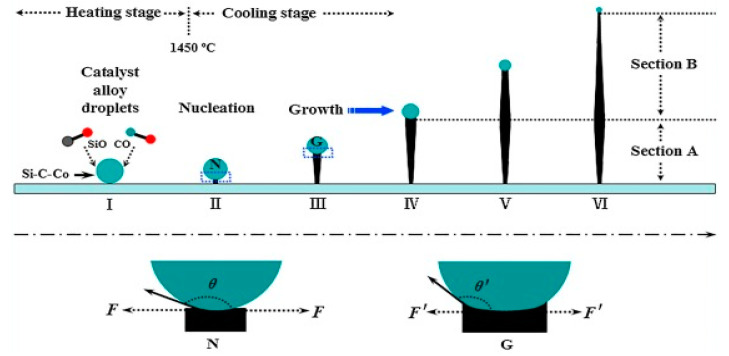
Overall formation mechanism of 1D SiC on the basis of the VLC method [56].

Different forms of 1D SiCs such as nanowires, nanobelts and/or nanoribbons, nanotubes, nanorods, and nanoneedles are widely fabricated by employing different synthetic techniques. Two widely accepted synthesis techniques for the synthesis of SiC NWs are gas- and solution-phases. The vapor-phase synthesis technique involves the formation of vapor during the reaction process that acts as a reactant itself to form a product [55]. CVD, combustion, heating, arc discharge, and pulsed-laser deposition are other widely accepted techniques. The NWs formation mechanism by the gas-phase synthetic technique can be further separated into vapor-liquid-solid (VLS) and vapor-solid (VS) mechanisms. The VLS mechanism possesses the ability of controlling or tuning the morphologies and orientation of NWs. However, obtained nanoarchitectures may not be in the purest form due to the presence of catalysts used. Therefore, this synthetic technique is employed as an alternative way to synthesize SiC NWs as indicated in Figure 5. 

Sun et al. used a high temperature CVD technique to fabricate β-SiC NWs [57]. In this study, Si-wafer, detonation shoot, and Fe were used as sources of Si, C, and catalyst, respectively. Moreover, the effect of temperature on morphologies was also investigated. At the low temperature of 1250 °C, SiC NWs were obtained, however, at the high temperature of 1300 and 1350 °C, the hexagonal nanocolumns and nanopyramids were obtained, respectively.

Feng et al. synthesized β-SiC NWs by heating Fe and polyureasilizane at 1500 °C [58]. The Fe and polyureasilizane acted as a catalyst and Si source, respectively. At the reaction temperature of 1500 °C, the polyureasilizane decomposed into SiO and CO and reacted with Fe to generate Fe-Si-C droplets. The as-generated Fe-Si-C droplets converted into NWs during the cooling process. The results showed that morphologies are controlled by adjusting cooling rates rather than heating rates.

**Figure 6:**
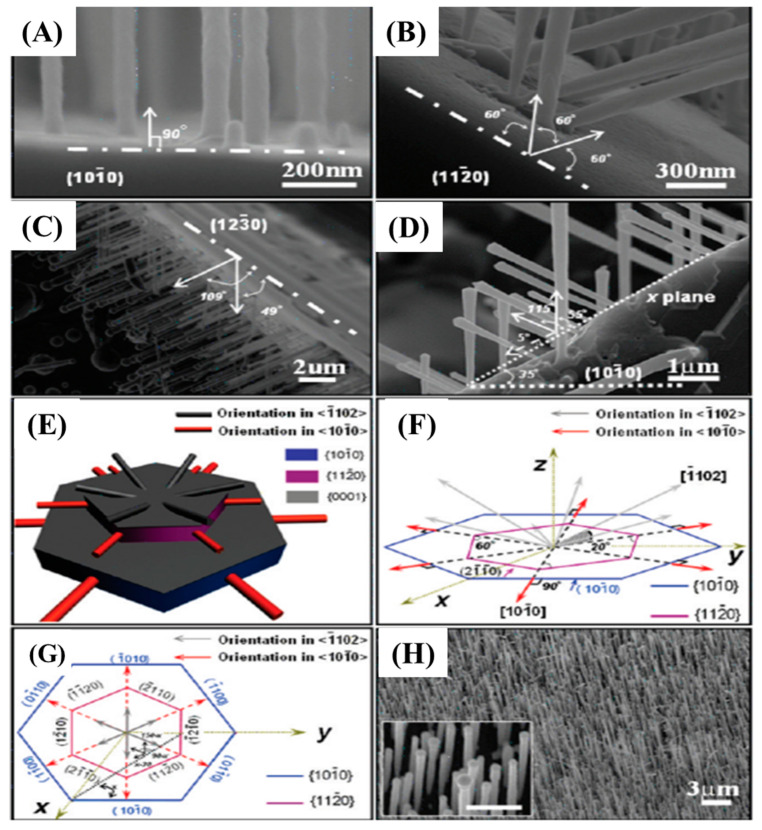
SEM images of SiC NWs grown on different SiC substrates (**A**–**C**). The dash and dot lines indicate the crystallographic planes of SiC substrates. SiC NWs were grown on x plane that is parallel to [0001] and with a tilted angle of 55 and 115° between the nanowire and substrate where x equals to 35° (**D**). The two dotted lines indicate x plane and tilt angle to X planes. A 3D model of NWs grown on SiC NWs on different planes (**E**). Pictorial representation indicating the relationships between substrate orientation and NWs axes (**F**,**G**). One well-aligned SiC NWs array fabricated on SiC substrate (**H**) [60].

Similarly, Lin et al. fabricated α-SiC (6H-SiC) nanorods through synthesis using a Fe-catalyzed arc-discharge technique. The graphite and SiC rods were employed as cathode and anode, respectively [59]. The Fe(NO)_3_ catalyzed 6H-SiC NWs on a single crystalline 6H-SiC was synthesized by Wang and co-workers as shown in Figure 6 [60]. 

In this experiment, they investigated the effect of substrate orientation (1010, 1220, and 1230) on the growth of SiC NWs. Perpendicular growth of well-aligned NWs was obtained on a (1010) substrate (Figure 6A,H). However, at 1220 and 1230 the NWs were grown at the tilt angles of 60, 120, 49, and 109°, respectively. Figure 6E–G represent the schematic representation of orientation growth of NWs at different tilt angles.

Recently, Liu et al. synthesized core-shell type SiC NWs using the CVD technique, which uses coconut cell as a carbon source and Si as a silicon source [61]. Coconut cell powder and Si powder were mixed together and heated at different temperatures of 1150, 1200, 1300, and 1400 °C for 3 h in an inert atmosphere. A large number of core-shell SiC NWs were obtained with lengths of 10–75 nm. The overall reaction was expressed as

Si (s) + O_2_ (g) → SiO_2_ (s)

2Si (s) + O_2_ (g) → 2SiO (g)

Si(s) + C(s) → SiC(g)

SiO(g) + 2C(s) → SiC(s) + CO(g)

SiO(g) + 3CO(g) → SiC(s) + 2CO_2_ (g)

3SiO(g) + Co(g) → SiC(s) + 2SiO_2_(s)

**Figure 7:**
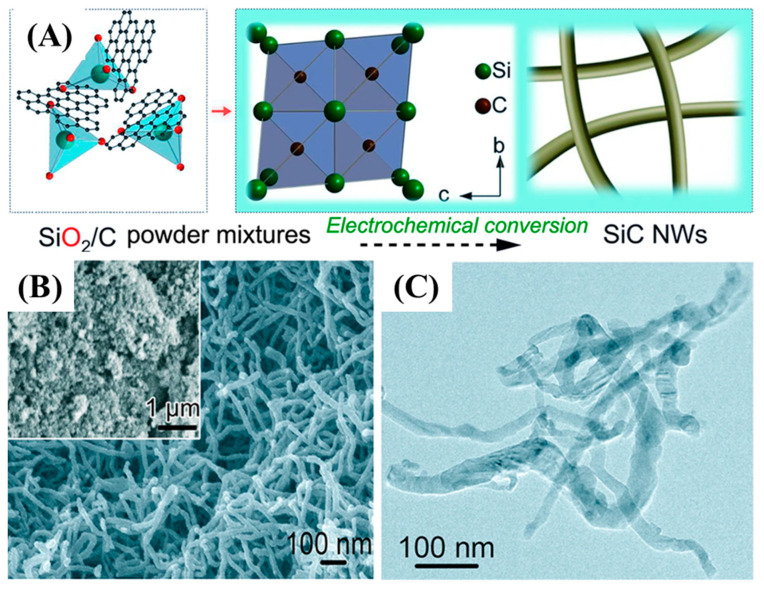
Overall synthesis process of SiC NWs (**A**), and FE-SEM images of SiC NWs (**B**,**C**) [62].

Zhou et al. synthesized SiC-CDC NWs via a molten salt solution (CaCl_2_) and a technique that useds SiO_2_ and C powders as precursors at 900 °C within and 3.1 V as indicated in Figure 7 [62]. 

Figure 7A shows the overall synthesis process of SiC NWs. The homogenous growth of SiC NWs was obtained with an average diameter of 30–50 nm and various micrometer in lengths as indicated by FE-SEM and TEM images. Teo et al. synthesized SiC nanotubes and NWs via a disproportionation reaction of silicon as Si-source with multiwalled carbon nanotubes as a template and source of carbon at 1250 °C for 40 min [63]. Zhang et al. synthesized 1D SiC NTs under hydrothermal conditions at a temperature of 470 °C and pressure of 8.0 MPa for 2 h. The as-synthesized SiC NTs were 8 nm in diameter and several hundred nanometers in length [64]. Similarly, Huu et al. employed the shape memory method using CNTs as a template to synthesize SiC NTs with a diameter of 100 nm and length of several tens of micrometers in length [65]. The other reported fabrication techniques of 1D SiC nanoarchitectures are summarized in Table 1. 

In addition, solution-based strategy is an alternative way to fabricate 1D SiC nanoarchitectures. Solvothermal and hydrothermal are the most widely used solution-based techniques because they are more convenient, economical, and time efficient. This method involves the crystallization of precursors present in the solution via reduction reactions. Ju et al. fabricated synthesized β-SiC NWs through a sulfur-assisted low temperature solvothermal method [81]. A mixture solution of Si, S, and C2Cl4 (tetrachlorethylene) was subject to the solvothermal method at 130 °C for 40 h. The overall reaction is expressed as:

2Si + S + C_2_C_l4_ + 6Na → 2SiC + Na_2_S + 4NaCl

The use of S source in the reaction system decreased the reaction potential barrier that permitted the formation of SiC NWs under suitable conditions. The obtained NWs were uniform with a diameter of 30 nm and length of 10 mm. Pei et al. synthesized β-SiC nanorods via the hydrothermal method at 470 °C under a pressure of 9.5 MPa [82]. In this report, they prepared large number of nanometer-sized SiC NWs with a length of 1 µm.

### 3.3. 2D SiC Nanoarchitectures 

2D architectures such as nanosheets, nanoflakes, and nanoplates have drawn intensive research interest in the context of a diverse field of applications such as electrochemical supercapacitors, sensors, solar cells, light emitting diods, and photocatalysis [83,84,85,86,87,88]. Several synthesis techniques (hydrothermal and/or solvothermal, carbothermal reduction, pyrolysis, and microwave plasma assisted CVD) have been employed to synthesize varieties of 2D nanoarchitectures [85,89,90,91]. 

**Figure 8:**
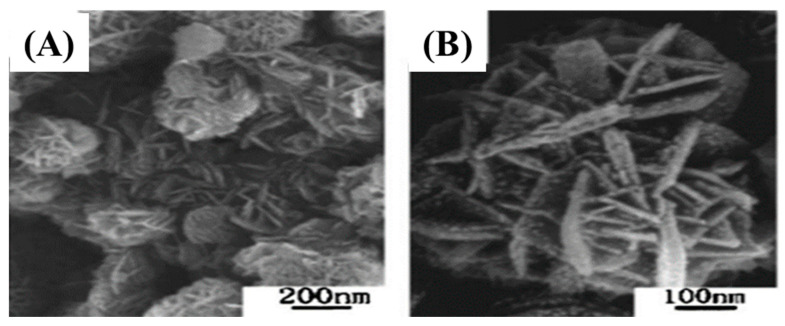
Low (**A**) and high (**B**) magnification FE-SEM images of 2D 2H-SiC nanoflakes [89].

Qian et al. synthesized 2D 2H-SiC nanoflakes through the low temperature solvothermal method for the first time. A mixture of CaC_2_ (1.5 g) and SiCl_4_ (8 mL) was poured in an autoclave, which was heated at 180 ℃ for 36 h [89]. The overall reaction and FESEM images (Figure 8) are given below

2CaC_2_ + SiC_l4_ → SiC + 2CaCl_2_ + 3C

Figure 8A,B represent the low and high magnification FE-SEM images of 2D 2H-SiC nanoflakes. The as-synthesized materials had a large number of nanoflakes with rough surfaces accumulated together (Figure 8A,B). The average diameter of individual nanoflakes were measured to be 200–500 nm and the diameter of 15 nm [89].

### 3.4. 3D SiC Nanoarchitectures

**Figure 9:**
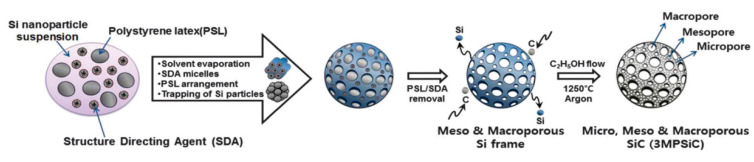
Schematic representation showing overall synthesis process [92].

Over the couple decades, huge breakthroughs have been achieved in designing, synthesizing, and evaluating 3D nanoarchitectures with tunable shapes and sizes. These materials are being widely investigated in diverse fields of applications such as gas sensors, electrode materials for supercapacitors, sensors, water-splitting, and photocatalysis. For instance, Kim et al. synthesized meso-, micro-, and macro-porous 3D SiC nanoarchitectures by using a template method followed by carbonization as presented in Figure 9 [92]. 

Further, Zho et al. fabricated ultralight and strong 3D SiCs via the carbothermal reduction of SiO with a graphene foam at 1550 °C [93]. SiC growth occurred through the solid-vapor mechanism via two steps. First, the SiO vapor filled the pores and reached the surfaces of the active carbon presented in the graphene network to form the SiC nuclei; the as-generated nuclei then converted in to SiC. The overall reaction mechanism was expressed as

SiO(g) + 2C(s) → SiC(s) + CO(g)

SiO(g) + 3CO(g) → SiC(s) + 2CO_2_(g)

## 4. Properties of SiC Nanoarchitectures

Due to their nanoscale-level structure, large surface-to-volume ratio, and quantum confinement effect, SiC nanoarchitectures possess different properties (mechanical, thermal, and electrical) than their bulk counterparts. 

### 4.1. Mechanical Properties

To be used in diverse fields of applications, SiC nanoarchitectures must have high mechanical properties such as high strength and hardness. These properties highly depend on the structure and dimensionality of materials. Wong et al. studied the fracture-strength of nanoarchitectured 1D SiC for the first time via atomic force microscopy (AFM) and had a maximum of 53.4 GPa due to a decreased number of defects [94]. In another report, Zang et al. tested the mechanical strength of β-SiC [111] NWs via in-situ TEM, which exhibited a plasticity property below room temperature [95]. Han et al. found flexibility in SiC NWs at ambient temperatures [96]. 

### 4.2. Thermal Properties

The thermal conductivity of SiC nanoarchitectures is negligible due to their low concentrations of electronic carriers; thermal conductivity is based on phonon transport. Lee et al. investigated the thermal conductivities of individual and double β-SiC NWs and found thermal conductivities of 82 ± 6 W/(mK) and 82 and 73 ± 5 W/(mK), respectively [97]. Similarly, Velentin et al. measured the thermal conductivities of β-SiC at different temperatures and found them to be 4–12 W/(mK) at temperatures 190–370 K [98].

### 4.3. Electrical Properties

SiC- is regarded as a p-type semiconductor due to its high-density donor states. The conductivities of SiC nanoarchitectures are always measured either by two-probe i-V (current–voltage) relation or three-terminal FET systems (Field effect transistors). Seong et al. studied the conductivity of SiC NWs using FET systems and found a resistivity of 2.2 × 10^−2^ Ω [99]. The conductivities of SiC nanostructures can be upgraded by synthesizing controllable crystalline structure, incorporation of dopants, and surface functionalizations [100,101,102].

### 4.4. Dielectric Property

SiCs possess excellent dielectric properties of low dielectric loss and temperature-stable dielectric permittivity. Wang et al. studied the dielectric properties of SiC ceramics and found dielectric behavior at temperatures above 600 K [103]. The dielectric property depends on the Si-C bod density, growth position, and ratio of Si and C. 

### 4.5. Wetting Property

1D SiC possesses a high wetting property. They are used to fabricate superhydrophobic surfaces due to their high surface roughness and outstanding stability. Niu et al. found that SiC NWs coated with organic perfluoroalkylsilane exhibited superhydrophobicity by reducing surface energy and increasing surface roughness [104,105]. Some other properties of SiC are summarized in Table 2.

## 5. Supercapacitor Applications

**Figure 10:**
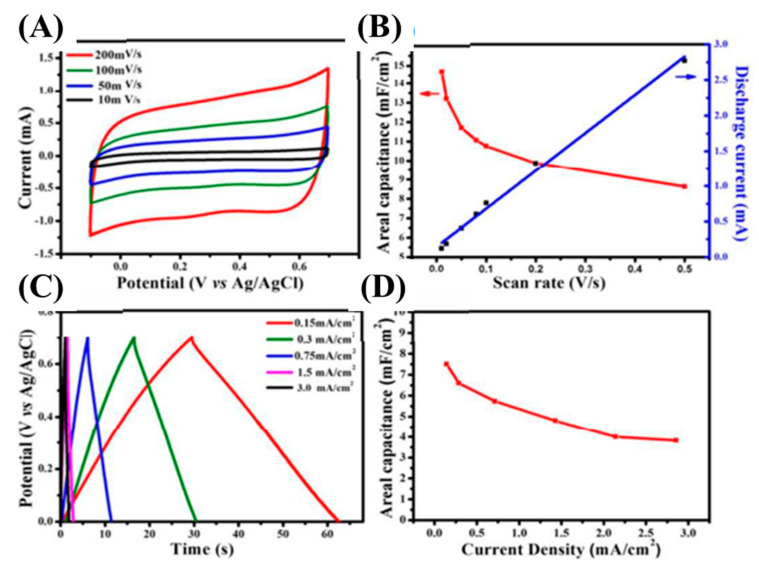
CV curves at various scan rates (**A**); relation between areal capacitances and discharge currents with different scan rates (**B**); GCD curves at different current densities (**C**); and relation between areal capacitances with different current densities (**D**) [106].

SiC-based nanoarchitectures and/or nanocomposites are considered to be promising electrode materials for electric double layer capacitors. SiC-based electrodes have realized excellent electrochemical performances in terms of their specific capacitance, cycle life time, and rate capability. This is due to their combability with different electrolytic environments. Supercapacitors’ electrode materials are generally fabricated with the apt architectures to upgrade their specific capacitances, conductivity, and reaction kinetics. Until now, plenty of studies have aimed to fully excavate their possibilities as supercapacitor electrodes. For instance, Mai et al. have developed a 4H-SiC nanochannels array for supercapacitors [106]. The electrochemical properties were investigated using CV and GCD techniques at an ambient temperature (Figure 10). Figure 10A shows the CV curves at different scan rates (10–200 mV/s). The rectangular shape at the low scan rate of 10 mV/s revealed typical EDLC characteristics. When the scan rate was increased from 10 to 200 mV/s, the CV curve retained its original shape, suggesting the high reversibility of the prepared electrode. The triangular GCD curves also revealed typical EDLC characteristics, which were well-matched with the CV curves (Figure 10C). The as-fabricated electrode delivered an outstanding areal capacitance of 14.8 mF/cm^2^ at the scan rate of 10 mV/s and retained 8.63 mF/cm^2^ even at the high scan rate of 200 mV/s, suggesting high-rate capability (Figure 10B). Moreover, its areal capacitances were also calculated using GCD curves and found to be 7.3 mF/cm^2^ at 0.15 mA/cm^2^, and 3.82 mF/cm^2^ at 3 mA/cm^2^, suggested a good rate capability (Figure 10D). As obtained, these outstanding electrochemical activities were due to the highly mesoporous SiC electrode being exposed to a highly active surface area that shortened the ion-electron diffusion paths.

Kim et al. fabricated micro-, meso-, and macro-porous 3D SiC frameworks using a simple template method followed by carbonization via aerosol spray and drying; the study investigated the potential applicability of supercapacitors [92]. The CV and GCD within the potential window of −1–0.9 V revealed EDLC behavior in 1 M neutral electrolytes. Moreover, the effect of the meso-porous size on the electrochemical activities was also investigated. The 3MPSiC-B electrode delivered the highest specific capacitance of 336.5 at a scan rate of 5 mV/s, as well as high conductivity, rate capability, and cyclic durability compared with the rest of the electrodes. These excellent electrochemical activates were mainly due to the presence of interconnected mesopores within the frameworks, as well as a high surface area and pore volume originating from multi-pores systems. 

**Figure 11:**
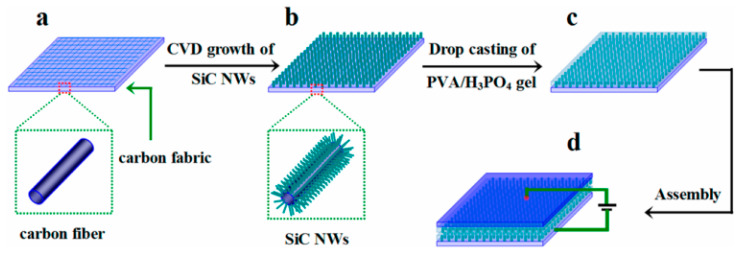
Overall synthesis process of SiC NWs@CF [107]. (**a**–**d**) indicate carbon fiber paper, SiC NWs@CF, PVA/H3PO4@SiC NWs@CF, and symmetric device, respectively.

**Figure 12:**
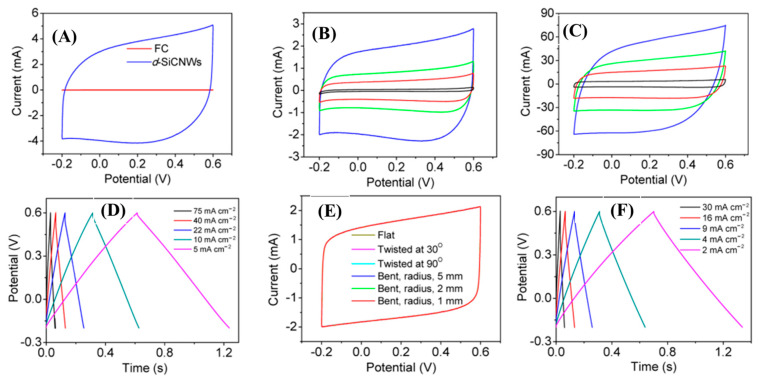
Electrochemical characterizations of N-doped SiC NWs: (**A**) CV comparative CV curves; (**B**,**C**) CV curves of pristine carbon cloths and SiC NWs at different scan rates (10–20,000 mV/s) within the potential window of −0.2–0.6 V; (**D**) GCD curves at different current densities (5–75 mA/cm^2^); (**E**,**F**) CV curves at different modes of deformation and GCD curves of all solid-state supercapacitors at various current densities (2–30 mA/cm^2^) [107].

Xie et al, designed nitrogen-doped 3C-SiC NWs on carbon fabric via the CVD technique, which was used as a freestanding electrode for supercapacitor applications [107]. First, N-doped 3C-SiC NWs were synthesized with different nitrogen contents using commercially available polysilazane as the source of Si, Co(No)_3_ as the catalyst, and flexible carbon as the substrate. This was heated at 1500 °C for 30 min in an ultrapure N_2_/Ar atmosphere at a flow rate of 200 sccm (Figure 11a–c). The well-defined N-doped NWs were equally distributed throughout the surface of carbon substrate; they had a diameter of 100–300 nm and an average length of 12 μm. The conductivity of nitrogen-doped d-SiC NWs (N-doped d-SiC) arrays was dramatically enhanced by the incorporation of an N dopant. Figure 12A–F shows the electrochemical test of the electrode material. From comparative CV curves in Figure 12A, pristine carbon cloth realized a negligible charge contribution. Figure 12B,C show the CV curves of pristine carbon cloths and N-doped d-SiC NWs electrodes at the scan rates of 10–20,000 mV/s. The CV curves at a low scan rate of 10 mV/s possessed a typical rectangular shape, suggesting EDLC behavior. Even at the ultra-high scan rate of 20,000 mV/s, the CV curves exhibited an identical shape, which highly suggests an outstanding reversibility. Figure 12D indicates triangular shaped GCD curves that were well-matched with the CV curves. High areal capacitances of 4.7 and 4.8 mF/cm^2^ were achieved in PVA-H_3_PO_4_ and KCL electrolytes. The outstanding electrochemical activities associated with the N-dopant, meso-, and macro-pores presented on the surface of NWs, and directly grown NWs on carbon cloths neglected the “dead mass” originating from the polymeric binders and conductive additives.

Yang et al. designed a free-standing SiC@graphitic carbon (SiC@C) composite as an outstanding supercapacitor electrode [108]. They used CVD technique to grow SiC NWs on the carbon fabric, similar to the previous report [107]. Then, an electrochemical deposition technique was employed to incorporate the carbon quantum dots, followed by calcination at 900 °C for 30 min in an inert atmosphere. The diameter of SiC NWs was measured to be 500 nm and grew uniformly on the carbon substrate. The as-designed electrode had a typical EDLC characteristic and specific capacitance of 78.98 mF/cm^2^ at a current density of 0.2 mA/cm^2^; this was 700% higher than that of pristine SiC NWs (9.56 mF/cm^2^), 94.1% cyclic durability after 10,000 GCD cycles, and outstanding electrical conductivity in 2M KCl electrolyte. An ionic liquid-based symmetric supercapacitor was developed with an energy density of 2.4 μWh/cm^2^ at power density of 65.1 μW/cm^2^. The outstanding electrochemical activities were due to the free-standing electrode materials, incorporation of quantum dots, and high-exposed electroactive active sites. 

**Figure 13:**
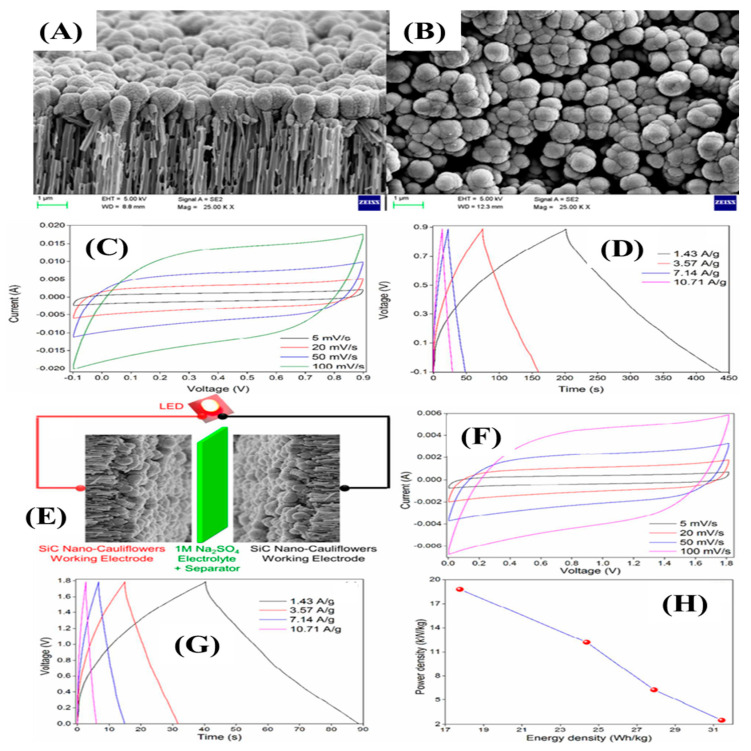
FE-SEM images (**A**,**B**); CV and GCD curves at various scan rates (5–100 mV/s) and current densities (1.43–10.71 A/g) (**C**,**D**); schematic representation of symmetric device (**E**); CV and GCD curves of symmetric device at various scan rates and current densities (**F**,**G**); ragone plot of the device (**H**) [109].

Chandra et al. synthesized SiC nano-cauliflowers on a silver-coated porous alumina substrate (OAA) through DC magnetron co-sputtering temperature at an ambient temperature [109]. The SiC nuclei formed and were deposited on the AAO surface, followed by the growth of the SiC nuclei, which took place in an island-like structure due to the slow diffusion and soaring interaction capacity of SiC adatoms by enduring the low surface energy. The radial and longitudinal directional growth took place by developing the grain boundaries and converting into cauliflower-shaped SiCs on an OAA surface. Figure 13A,B indicates the cross-section and top view of the resultant materials. The thickness of the SiC layer on the OAA surface was 100 nm. The orientation and arrangement of the nanostructure was in the shape of a cauliflower. The electrochemical tests for the supercapacitors electrode were executed in aqueous electrolyte (Na_2_SO_4_). Figure 13C,D indicate the CV and GCD curves, which show the typical EDLC characteristics and high reversibility of the SiC electrode. The highest specific capacitances of 300 F/g and 283 F/g were obtained at a low scan rate of 5 mV/s and low current density of 1.43 A/g; they retained the capacitance values of 150 F/g and 139 F/g at the scan rate of 100 mV/s and at 10.71 A/g, respectively, indicating an enticing rate capability. Such high specific capacitances and rate capabilities were due to the 3D nanostructures that provided a huge number of exposed electroactive sites. In addition, a high potential window of symmetric supercapacitors was constructed (Figure 13E). The CV and GCD curves also obeyed the EDLC charge storage process within the potential window of 0–1.8 V. The large operating potential window of 1.8 V was due to the strong solvation of energy of the Na^+^ ions and SO_4_^−^ [30]. The symmetric device had an energy density of 31.43 Wh/kg at a power density of 2.5 kW/kg, indicated by the ragone plot in Figure 12H. 

In other work, Jiang et al.’s naocrystalline, microcryatalline, and epitaxial 3C-SiC films on mono-crystalline SiC substrate via microwave CVD technique and their detailed electrochemical activities were investigated in 1 M H_2_SO_4_ electrolytes [110]. The electrochemical results revealed that epitaxial 3C-SiC had the lowest EDLC capacitance but the highest reversibility, and nanocrystalline 3C-SiC had the highest capacitance and low reversibility due to its structural homogeneity. Maboudian et al. developed SiC NWs base micro-supercapacitors [111]. 3C-SiC NWs were grown on Si(100) substrate with a SiO_2_ isolation layer through a nickel-catalyzed low-pressure CVD process. Dense and homogenous growth of NWs occurred on the surface of the Si-substrate with a thickness of 6 μm. 3C-SiC NWs showed perfect EDLC characteristics within the working potential window of −0.2–0.6 V, had a specific capacitance of 240 μF/cm^2^ at a current density of 100 mV/s, and had a capacitance retention of 95% after 2 × 10^5^ cycles. Yang et al. developed SiC nanofibers on carbon fabric as a supercapacitor electrode, which delivered an outstanding areal capacitance of 4.36 mF/cm^2^ at a scan rate of 0.05 V/s, and splendid cyclic durability of 106.51% after 10,000 cycles in KCl electrolyte [83]. Lust et al. synthesized carbon-dioxide -activated SiC-CDC that possessed the specific capacitance of 125 F/g at 1 mA/cm^2^ in the organic electrolyte (C2H5)3CH3NBF4 solution in acetonitrile [112]. Kim et al. synthesized a porous SiC carbothermal reduction reaction at 1250 °C, which possessed a high specific capacitance of 336.5 F/g and a high rate capability of 90.3 % from 5 to 500 mV/s in neutral electrolyte [92]. Ma et al. synthesized SiC/Pyrrolic-N doped carbon for a supercapacitor electrode. The as-synthesized electrode delivered a high specific capacitance of 369 F/g at 0.5 A/g with 100 % capacitance retention after 5000 cycles [113]. Bhargava et al. reported SiC NWs around 3–4 μm in length and 200–400 nm in diameter with capacitances of 96 μF/cm^2^ [114]. Kim et al. synthesized porous SiC flakes from discarded silicon wafer via a carbonization process at 1250 °C [115]. The porous SiC nanoflakes had ideal EDLC behavior and specific capacitances of 49.2 F/g and 38.7 F/g at scan rate of 5 mV/s in 1M KCl and 1 M BMIM BF4/AN, electrolytes, respectively. Further, the energy density of 65.84 Wh/g was achieved with outstanding cyclic durability of 98.65% after 20,000 cycles. Similarly, Kim et al. produced β-polytype porous SiC nanospheres by heating at 80 °C for 18 h in a water-cooled condenser [116]. The resultant β-polytype porous SiC nanospheres had a typical EDLC behavior with an outstanding specific capacitance of 82.9 and 60.3 F/g in KCl, and TEABF_4_/AN electrolytes at scan rate of 5 mV/s, respectively. The device possessed ultra-high energy density of 102.59 Wh/kg in TEABF_4_/AN electrolyte. 

**Figure 14:**
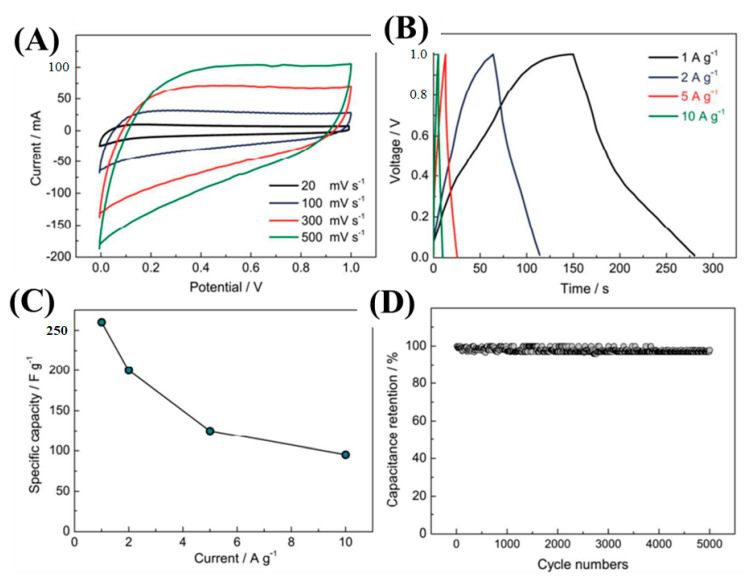
Electrochemical investigations: CV and GCD curves (**A**,**B**); plot between specific capacity versus current density (**C**); and cyclic durability test (**D**) [117].

Lu et al. fabricated SiC-NWs-derived-carbon NWs (SiC NWs-CDC) via a molten salt electrochemical process [117]. The as-synthesized SiC-CDC NWs were used as electrode materials for supercapacitor applications. CV and GCD techniques were employed to investigate the electrochemical activities. Figure 14A showes the CV curves at different scan rates of 20–500 mV/s within the potential window of 0.0–1.0 V in 6 M KOH solution. The CV curves had perfect EDLC characteristics. As the scan rate increased, the integral CV curves also increased without deformation, indicating the high reversibility of the SiC-CDC NWs electrode. The GCD curves were also executed to evaluate the specific capacitances (Figure 14B). From the GCD curves, a specific capacitance of 256 and 95 F/g at 1 and 10 A/g current densities, respectively, were achieved (Figure 14C). Furthermore, the cyclic durability of the electrode materials was carried out and had a 97.9% capacity retention after 5000 GCD cycles (Figure 14D). The outstanding electrochemical activities were mainly due to the presence of dual-scale porous structures obtained during the molten-salt etching process. 1D NWs were beneficial for electron transport, and dual-scale nano-architectured SiC-CDC NWs shortened the electron diffusion path.

Hou et al. employed the mild fabrication technique for the synthesis of carbon-coated SiC nanosheets (SiC/C) and employed as supercapacitor applications. In this paper, the authors used a facile and mild wet-chemical etching technique to fabricate SiC/C using carbon aluminum silicate (Al_4_SiC_4_) as raw material. Hydrofluoric acid (HF) was used as the etching agent; it broke down the C-Al bond that led to the formation of SiC nanosheets. The as-synthesized nanosheets possessed a smooth and rectangular shape that was 150 nm in width, 500 nm in length, and 10 nm in thickness. Moreover, the electrochemical activities of the synthesized materials were investigated in the 1 M Na_2_SO_4_ electrolyte within the potential window of 0.0–0.6 V. Within the working potential window, it had typical EDLC characteristics and areal capacitance of 734 μF/cm^2^ at a scan rate of 10 mV/s (130 F/g). Even at the high scan rate of 500 mV/s, it retained 46.8% of its original capacitance and 91% of its capacitance after 20,000 cycles. The outstanding electrochemical activities can be attributed to the 3C/2H-SiC heterojunctions and presence of C-layers [118]. Fang et al. fabricated all-solid-state one-chip supercapacitors using 4H-SiC NWs arrays. The as-fabricated 4H-SiC NWs arrays electrode delivered a record-breaking areal capacitance of 23.6 mF/cm^2^ at a scan rate of 10 mV/s, a capacitance retention of 51.2% when the scan rate was increased to 1500 mV/s, and 94.8% cyclic durability after 10,000 cycles. Moreover, all-solid-state one chip supercapacitors delivered an energy density of 5.2 μWh/cm^2^ at a power density of 11.2 mW/cm^2^. These outstanding electrochemical activities can be attributed to the binder-free electrode, the outstanding physiochemical properties of SiC, and the SiC NWs unique architectures [119]. Table 3 represents the electrochemical activities of SiC-based electrode materials.

## 6. Conclusions and Outlooks

This review summarized recent achievements in SiC-based nanoarchitecture and detailed their synthesis process, properties, and supercapacitors applications. Varieties of SiC nanoarchitectures were synthesized using different synthetic techniques to tune their structures, morphologies, dimensions, electrochemical activities, and their potential applicability. The synthetic techniques employed so far are expensive, time-consuming, and lack sufficient growth mechanisms. Simple and cost-effective fabrication techniques are required, as is a better understanding of the kinetic and thermodynamic growth of SiC nanoarchitectures that can be helpful in controlling their morphologies, dimensions, and orientations.

SiC-based electrode materials still suffer from low electrochemical activities. Increasing their electrochemical activities is the most important task of a supercapacitor’s design. The low electrochemical activities of SiC-based electrode materials can be increased by combining them with some traditional and battery-type counterparts. Strategies to incorporate new innovative materials may open new avenues into high-performance SiC-based supercapacitors.

Overall: SiC-based electrode materials for supercapacitors remains a research gap to investigate. High-performance SiC-based supercapacitors can thrive through future research that would aim to establish a comprehensive understanding of electrode materials design, fabrication techniques, and the making of composites and doping of n/p-types dopants.

## Figures and Tables

**Table 1:** Fabrication techniques of 1D SiC nanoarchitectures.

Nanoarchitetures	Fabrication Techniques	Temperature (°C)	Ref.
β-SiC NWs	Heating	1300	[66]
β-SiC NWs	CVD	1250	[67]
β-SiC NWs	Carbothermal reduction	1500	[68]
β-SiC NWs	Electrospinning followed by carbothermal reduction	1560	[69]
Twined β-SiC NWs	CVD	370	[70]
Hexagonal β-SiC nanoprisms	Thermal evaporation	1450	[71]
β-SiC nanocones	CVD	1300	[72]
SiC nanorods	CNTs confined heating	1200	[73]
α-SiC nanorods	Arc-plasma	2700	[74]
6H-SiC	Carbothermal reduction	1300–1400	[75]
β-SiC NWS	Thermal evaporation	1550	[76]
Inverted β-SiC NWs	Heating	1800	[77]
SiC NWs	heating	1460	[55]
β-SiC NWs	Carbothermal reduction	1300	[78]
3C-SiC NWs	CVD	1000	[79]
N-doped 3C-SiC nanobelts	CVD	1500	[80]

**Table 2:** Properties of SiC.

Mechanical Properties	SI/Metric (Imperial)	SI/Metric	Imperial
Density	Gm/cc	3.1	193.5
Porosity	%	0	0
Color		Black	
Flexural Strength	MPa	550	80
Elastic Modulus	GPa	410	59.5
Compressive Strength	MPa	3900	566
Shear Modulus	GPa		
Bulk Modulus	GPa		
Poisson’s Ratio		0.14	
Hardness	Kg/mm^2^	2800	
Fracture Toughness	MPa·m^1/2^	4.6	-
Maximum Use Temperature	°C (°F)	1650	3800
**Electric Properties**			
Dielectric Strength	Volt/mil		Semiconductor
Volume Resistivity	Ohm·cm		Dopant dependent
**Thermal Properties**			
Thermal Conductivity	W/m·°K	120	
Coffecient of Thermal Expression	10^−6^/°C	4	
Specific Heat	J/Kg·°K	750	

**Table 3:** Some SiC-based electrode materials and their electrochemical activities.

Electrode Materials	Electrolyte	Specific Capacitance	References
4H-SiC NWs arrays	2 M KCl	22.3 mF/cm^2^ at 10 mV/s	[119]
Boron doped SiC	1 M H_2_SO_4_	232 F/g at 2.2 A/g	[120]
4H-SiC NWs arrays	2 M KCl	14.8 F/cm^3^ at 10 mV/s	[119]
SiC@PANI core/shell	1 M Na_2_SO_4_	352 mF/cm^2^ at 1 mA/cm^2^	[121]
SiC NWs@CNTs@NiCO_2_O_4_	1 M KOH	2302 F/g at 1 A/g	[122]
NiSi/SiC core-shell NWs	1 M KOH	234.13 mF/cm^2^ at 1 mA/cm^2^	[123]
HPC/SiC	1 M Na_2_SO_4_	234.2 F/g at 1 A/g	[124]
SiC NWs@MoS_2_	1 M Na_2_SO_4_	200.35 F/g at 0.1 A/g	[125]
SiC/B-MnOx	1 M Na_2_SO_4_	251.3 F/g at 10 mV/s	[126]
SiC NWs	3.5 M KCl	1.7 μF/cm^2^ at 50 mV/s	[127]
SiC NWs@C		3.3 mF/cm^2^ at 20 mV/s	[114]
SiC NWs@PEDOT	0.1 M TBA BF_4_	50 F/g at 100 mV/s	[128]
Ru@SiC NWs	1 M Na_2_SO_4_	36.25 mF/cm^2^ at 5 mV/s	[129]
SiC@CF	2 M KCl	23 mF/cm^2^ at 50 mV/s	[130]
C@SiC	6 M KOH	220 F/g at 1 A/g	[131]
SiC NWs@Ni(OH)_2_	1 M KOH	1724 F/g at 2 A/g	[132]
SiC NWs@GP	0.1 M H_2_SO_4_	25.6 mF/cm^2^ at 0.2 A/cm^2^	[133]
Eesoporous SiC-CDC	0.5 M NEtBF_4_/AC	80 F/g at 1 V/s	[134]
MnO_2_@SiC	1 M Na_2_SO_4_	273.2 F/g at 10 mV/s	[135]
SiC@Fe_3_O_4_	1 M KOH	423.2 F/g	[136]

## Data Availability

Not applicable.
